# Topological Optimization of Auxetic Coronary Stents Considering Hemodynamics

**DOI:** 10.3389/fbioe.2021.728914

**Published:** 2021-09-13

**Authors:** Huipeng Xue, Suvash C. Saha, Susann Beier, Nigel Jepson, Zhen Luo

**Affiliations:** ^1^ School of Mechanical and Mechatronic Engineering, University of Technology Sydney, Sydney, NSW, Australia; ^2^ School of Mechanical and Manufacturing Engineering, University of New South Wales, Kensington, NSW, Australia; ^3^ Department Cardiology, Prince of Wales Hospital, Randwick, NSW, Australia

**Keywords:** coronary stents, topology optimization, auxetic metamaterials, hemodynamics, computational fluid dynamics

## Abstract

This paper is to design a new type of auxetic metamaterial-inspired structural architectures to innovate coronary stents under hemodynamics via a topological optimization method. The new architectures will low the occurrence of stent thrombosis (ST) and in-stent restenosis (ISR) associated with the mechanical factors and the adverse hemodynamics. A multiscale level-set approach with the numerical homogenization method and computational fluid dynamics is applied to implement auxetic microarchitectures and stenting structure. A homogenized effective modified fluid permeability (MFP) is proposed to efficiently connect design variables with motions of blood flow around the stent, and a Darcy-Stokes system is used to describe the coupling behavior of the stent structure and fluid. The optimization is formulated to include three objectives from different scales: MFP and auxetic property in the microscale and stenting stiffness in the macroscale. The design is numerically validated in the commercial software MATLAB and ANSYS, respectively. The simulation results show that the new design can not only supply desired auxetic behavior to benefit the deliverability and reduce incidence of the mechanical failure but also improve wall shear stress distribution to low the induced adverse hemodynamic changes. Hence, the proposed stenting architectures can help improve safety in stent implantation, to facilitate design of new generation of stents.

## Introduction

Percutaneous coronary intervention (PCI) has been popular as a common treatment for coronary artery disease, but the risks of stent thrombosis (ST) and in-stent restenosis (ISR) still threaten the safety of stent implantation and represent a serious clinical shortfall. Although various reasons accounting for these adverse biological responses that have not been fully understood, the stent essentially serves as a mechanical structure and is believed to have a major effect on the ST and ISR complications. In our previous work (Xue et al., 2020), the self-expanding (SE) auxetic stent has been demonstrated to have capability in supplying adaptive deformation to help overcome the mechanical failures and low the incidence of the ST and ISR complications. In this research, the stent-induced hemodynamic changes will also be combined into stents to further improve stenting performance from both mechanical and hemodynamic aspects, because the stenting structures are primally influenced not only by mechanical failure including inadequate expansion, fracture, malapposition, foreshortening and dogbone, but also by the hemodynamic changes induced in stent implantation.

The adverse hemodynamic changes caused by implanting stents are also related to high risks of ISR and ST (Tomaszewski et al., 2020; Gharleghi et al., 2021). In 1995, Rogers and Edelman (1995) noted that the stenting structures might affect restenosis and thrombosis in the arteries through actions other than the mechanical injures. Then, neointimal hyperplasia independent of arterial injury was first investigated in stented rabbit iliac arteries (Garasic et al., 2000). The results indicated that stenting structures might adversely affect fluid motions in the arterial lumen and induce intimal thicken. Nearly in the same period, Berry et al. (2000) shown that enlarging strut spacings can facilitate blood flow and reduce the cumulation of neointima in a stented coronary artery. Then, the inverse proportion relationship between neointimal hyperplasia and the change of wall shear stress (WSS) after stent implantation was found by Wentzel et al. (2001) and further studied by LaDisa et al. (2005).

In hemodynamics, the WSS on the endothelial cells of coronary arteries has been demonstrated as a significant factor connected with atherosclerotic disease development (Ku, 1997). The protrusions of stent struts inside the arterial lumen disturb blood flow, so as to change the local flow environment, and further affect the distributions of wall stress in the stented segment. The shear stress is generated when blood flows through the endothelium, which is proportional to the blood viscosity and the velocity gradient. The WSS is related to the vascular hemodynamics and correlated with radial responses, intimal thickening, and platelet thrombosis (Gijsen et al., 2019). The WSS under a steady laminar flow can promote endothelial cells to release factors to inhibit coagulation, migration of leukocytes, and smooth muscle proliferation, to benefit the healing of narrowed vessels (Traub and Berk, 1998). However, unusual WSS can cause neointimal hyperplasia and atherosclerotic plaque formation. Many studies have shown that the areas with WSS lower than 0.5 Pa are prone to intimal thickening and atherosclerosis, while the WSS higher than 2.5 Pa may increase the risk of plaque rupture and thrombosis (Beier et al., 2016; Gharleghi et al., 2021). The distributions of WSS will change after implanting stents due to the altered velocity profile of the blood flow in the stented segment (Gijsen et al., 2019). Therefore, WSS has been widely adopted as a metric to evaluate the impact of stent implantation on hemodynamic changes. Sometimes, for nonuniform flow and pulsatile flow, wall shear stress gradient (WSSG) and time-averaged wall shear stress (TAWSS) are also used to evaluate the stent, respectively (Gharleghi et al., 2019). Although the stent induced hemodynamic changes can be quantified via the WSS distribution, how the stenting structures affect the WSS in the stented segment is still unclear.

We can find that the stent protrusion is related to the WSS and affects the adverse hemodynamic. The influence of the stent protrusion can be reduced but it is inevitable in stent implantation. For instance, the flow disturbance can be eliminated by fully embedding stent architectures into artery walls (Gharleghi et al., 2019). However, by doing this, the over-sized stent can lead to high tension stress on the vessel wall and may cause serious injury (Nolan and Lally, 2018). As a result, the influences of strut geometric characteristics on flow patterns have been widely investigated (Putra et al., 2019; Wei et al., 2019; Gharleghi et al., 2021) to minimize the adverse hemodynamic effect on stents via designs. Among stenting geometric characteristics, strut thickness contributes the most concern because it directly determines the degree of stent protrusion and is strongly connected to hemodynamic changes. Thicker struts were found to significantly promote intimal thickening (Stiehm et al., 2019). Strut spacing is another factor, as a large spacing can restore disturbed flow (Bekal et al., 2018). Since the adverse hemodynamic changes are usually presented as flow disturbance, streamlined stent strut cross-section profiles were suggested to facilitate blood flow (Melzer et al., 2019). Also, varying strut angles (Beier et al., 2016) were also found to benefit hemodynamic designs, such as aligning the orientations of struts and connectors with the flow direction (Bekal et al., 2018).

Most current stent designs usually utilize empirical or surrogate model-based methods to study the influence of variables, such as struts thickness, width, angle, and spacings on WSS. The earlier works were mainly focused on a single objective, such as minimizing low WSS areas (Gundert et al., 2012). However, the stent usually works in a complex environment associated with structural mechanics and fluid dynamics (Bressloff et al., 2016). Thus, several studies have tried to perform design by accounting for both aspects, simultaneously. For example in the work (Putra et al., 2019) the design objectives were defined based on both mechanical and hemodynamic metrics, including recoil, flexibility, and WSS. Objectives related to drug release were also taken into account in stent design (Vijayaratnam et al., 2019). However, these optimization formulations mostly adopt a single objective to consider hemodynamic and a small number of structural design variables related to sizes, which in practice are hard to capture the impacts of stenting structures on the blood flow alterations in the arteries. Hence, two objectives, such as mean square WSS and mean swirl value (Doutel et al., 2018), or recirculation zone length and struts reattachment distance (Gharleghi et al., 2019) have been defined to implement the design, although the improvements were limited. After that, researchers attempted to include more parameters with strut geometric characteristics into the optimization (Gharleghi et al., 2021), while too many variables and multiple design objectives lead to very complex optimization formulations.

The above designs narrow the freedom to generate new stents by only allowing the change of stent geometrical parameters. Particularly, the conventional designs are not able to change structural topologies and shapes for uncovering novel architectures to improve stenting performance. Hence, new design methodologies are in demand. Recently, topology optimization is experiencing popularity as an efficient tool for systematic design of new structures. Since stents subject to hemodynamic changes are sensitive to both the shape and topology of stenting structures, this paper employs a parametric level set method (PLSM) (Luo et al., 2007; Luo et al., 2009) to implement topological design of stents. PLSM can generate structures with clear and smooth boundaries, more suitable for hemodynamic stent designs. PLSM has been successfully applied designs of mechanical metamaterials and auxetic structures (Wang et al., 2014; Wu et al., 2017; Li et al., 2018). It is noted that a stent usually consists of a number of uniform and repetitive structural architectures. In this paper the numerical homogenization method is used to evaluate effective properties (e.g., the elasticity tensor) of underline microstructures. This paper integrates the numerical homogenization method and Computational fluid dynamics (Gundert et al., 2012) with the PLSM approach (Luo et al., 2007) to fulfill topological design of stents using auxetic metamaterials. Auxetics are engineered mechanical metamaterials consisting of periodic microstructures with effective negative Poisson’s ratio (NPR) (Lakes, 1993), exhibiting counterintuitive property in deforming structures: contract or expand in a transverse direction when compressed or stretched in an axial direction. Such behaviour in elastic deformation will considerably benefit stents, e.g., energy absorption, adaptive stiffness, indentation resistance, and enhanced fracture toughness, which gives the stenting structures enhanced flexibility and adaptability to automatically match the artery shape during the deformation.

A modified fluid permeability (MFP) is proposed to quantify obstructions to fluid at different directions in stenting microstructures, to include the hemodynamic changes into stent design to describe the stent-induced fluid changes. A fluid permeability is often used to quantify the ability of a porous medium to allow fluids passing through it. It is mainly determined by the porosity, shapes of pores, and their distributions. The porous medium with higher permeability can enable the fluid to easily move through it. Implanting stent changes flow environments due to the obstructions from stent struts (Putra et al., 2019). The obstructions can cause flow disturbances, flow separation, recirculation zones, resulting in adverse clinical outcomes (Bukač et al., 2019). Then, reducing the adverse hemodynamic changes around stents can be obtained by minimizing the MFP in directions of blood flow.

## Assumptions in Stent Designs

To perform a hemodynamic topology optimization for the stent, the simulation of blood flow in the stented artery is necessary. The complex endovascular flow environment and the interactions between blood, stent, and endothelial cells are challenging in practice. Although CFD provides an efficient way to describe fluid motion by solving Naiver-Stokes (NS) equations, a complex blood flow model still leads to a high computational cost in solving the full NS equations. Some assumptions necessary to simply the fluid computation and facilitate the design optimization are given as follows.

### Blood Flow

Blood is an incompressible fluid, so the NS equations with incompressible conditions are utilized to describe the motion of blood flow. Eq. 1 is the momentum equation, Eq. 2 is the incompressibility condition, and Eq. 3 is the no-slip condition on the boundary.

$$\rho \frac{\partial u}{\partial t}+\rho \left(u\cdot \nabla \right)u-\mu \Delta u=-\nabla p+\rho g\;\;\;\;\;\;\;\text{in}\;{\text{Ω}}_{\text{f}}$$ (1)

$$\nabla \cdot u=0\;\;\;\;\;\;\;\text{in}\;{\text{Ω}}_{\text{f}}$$ (2)

$$u=0\;\;\;\;\;\;\;\text{on }\;{\text{Γ}}_{\text{f}}$$ (3)

where Ω_f_ is a fluid domain with boundary Γ_f_ *. ρ* and *μ* denote fluid density and viscosity, respectively. **u** is the flow velocity, *t* is time, *p* is the pressure, and **g** is body accelerations acting on the fluid, such as gravity and inertial. *ρ* **g** denotes the external body force vector including inertia forces. ∇, Δ and ∇⋅ are the gradient, Laplacian, and divergence operators, respectively. The assumptions are:

1) Geometric assumptions: the stent is assumed as a straight, rigid body with constant thickness and uniformed horizontal cross-section profile, and no stent deformations are generated under the blood flow. These assumptions will reasonably simplify the CFD model (Balossino et al., 2008).
2) Blood flow is assumed as an incompressible Newtonian fluid with a steady flow state, so the unsteady item in Eq. 1 is neglected. Based on the study of (Johnston et al., 2006), only about 30% cardiac cycle was obviously affected by the non-Newtonian model. Blood flows in all arteries present pulsatile motions due to the cyclic behaviors of systole and diastole (Ku, 1997). Hence, the blood flow with a steady-state (Mattace-Raso et al., 2006) is usually adopted, and the inlet velocity is defined as a time-averaged value over a cardiac cycle.
3) The optimization intends to improve the blood flow in this worst region near the artery walls. Since the blood flow is relatively slow near the artery walls, the convective and inertia items can be ignored. Hence, the blood flow is consequently simplified to a Stokes flow in the computational domain, where the momentum equations are given in Eq. 4.

$$\begin{array}{l} -\mu \Delta u=-\nabla p+\rho g\;\;\;\;\;\;\;\text{in}\;{\text{Ω}}_{\text{f}}\\ \nabla \cdot u=0\;\;\;\;\;\;\;\text{in}\;{\text{Ω}}_{\text{f}}\\ u=0\;\;\;\;\;\;\;\text{on}\;{\text{Γ}}_{\text{f}} \end{array}$$ (4)

### Design Domain

**FIGURE 1:**
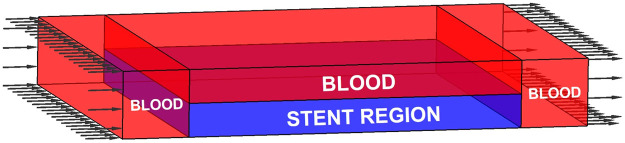
The 3D computational domain.

In the stented segment of the artery, blood flows through the inner lumen and fills the gaps between stent struts. The fluid is separated by the stent and divided into different regions. A 3D computational domain based on one stenting microstructure is established for the CFD model, as shown in Figure 1. The domain has four regions: three blood regions with red colour and one stent region with blue colour. In the middle of the computational domain, two thin layers with the same thicknesses are defined as the stenting microstructures, with one microstructure defined in the stent region at the bottom layer. Blood flows through the whole top layer and fills the gaps between stent struts in the bottom layer. Hence, the stent region consists of one stent microstructure filled with the blood in gaps. Two blood regions with the same sizes are defined in the two ends of the microstructure to avoid the impacts of the flow boundaries. Since the fluid convection is ignored, various lengths of inlet and outlet will affect the fluid. The CFD simulation is then performed under the following conditions:

1) A constant inlet velocity of blood flow specified on the left side of the computational domain.
2) The outlet boundary with nearly zero pressure located on the right side.
3) No-slip conditions applied on the outside surfaces of the simulation region.
4) No additional conditions to the solid-fluid interfaces due to the restriction to the flow in solid areas.

**FIGURE 2:**
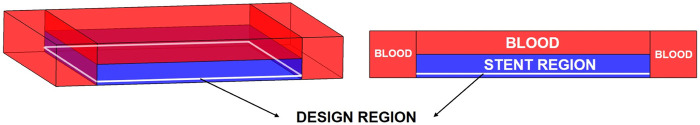
The 2D design domain.

As the assumption of constant thickness, a 2D horizontal cross-section profile is extracted from a stent microstructure and defined as the design domain. The outer contours of the design domain are presented with white lines, and the bottom of the stent region is defined as the artery wall, as given in Figure 2. The velocities at the design domain are extracted to evaluate the index MFP. After that, the design variables are updated by the optimization method. Then, the material layout in the stent region can be correspondingly updated by the new microstructural profile. In this way, the associations between the computation and design domains are established.

## Parametric Level Set Method

**FIGURE 3:**
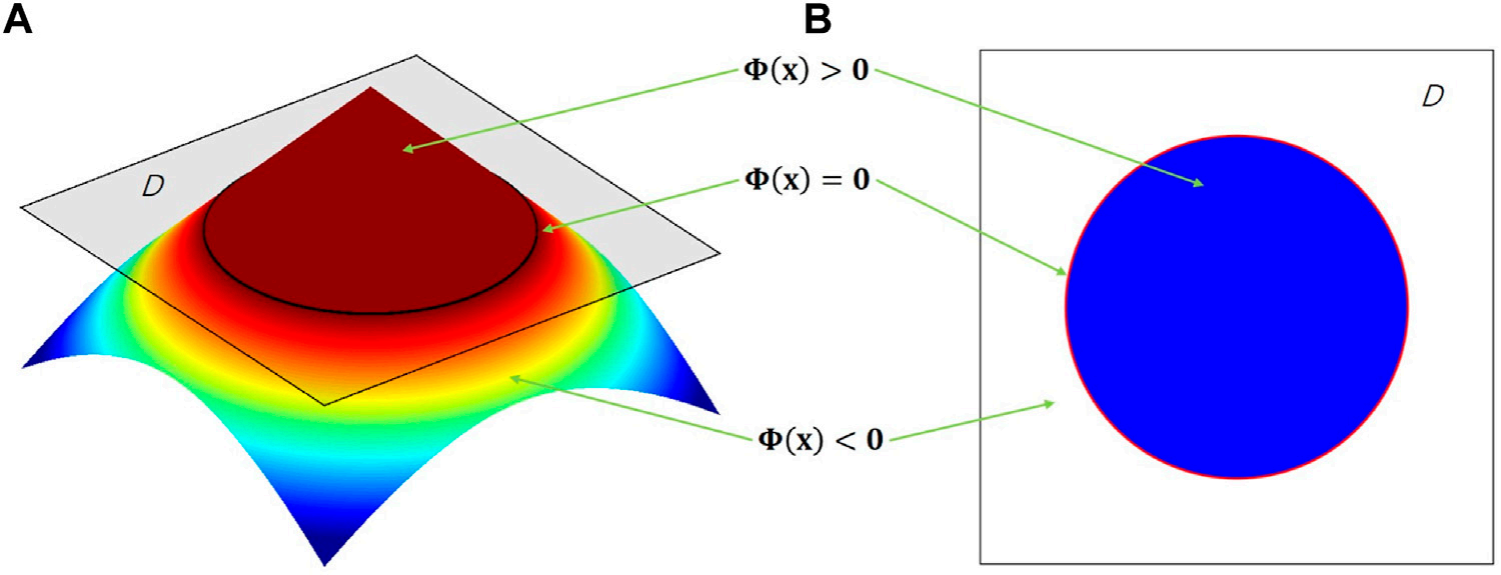
**(A)** 3D level set surface; 2D level set boundary.

LSM implicitly defines the structural boundary as the zero-level set of a higher-dimensional scalar function *Φ* (*x*). For example, a two-dimensional (2D) design, given in Figure 3 **,** is defined by

$$\left\{ \begin{array}{l} \Phi \left(x\right)>0\;\;\;\;\;\;x\in \Omega \backslash \partial \Omega \;\;\;\;\;\;\;\;\;\;\;\;\;\;\;\left(\text{Solid}\right)\\ \Phi \left(x\right)=0\;\;\;\;\;x\in \partial \Omega \;\;\;\;\;\;\;\;\;\;\;\;\;\;\;\;\;\;\left(\text{Boundary}\right)\\ \Phi \left(x\right)<0\;\;\;\;\;\;x\in D\backslash \left(\Omega \cup \partial \Omega \right)\;\;\;\;\;\;\;\;\;\;\;\;\left(\text{Void}\right) \end{array}\right.$$ (5)

where *x* is a point in space *D*. *Ω* and *∂Ω* are the design domain and its boundary, respectively. The dynamic motion of the level set function then drives the topological shape changes of the structure. Differentiating zero level set function with respect to a pseudo time *t* leads to motion of interface as

$$\frac{\partial \Phi \left(x,t\right)}{\partial t}-v_{n}\left|\nabla \Phi \left(x,t\right)\right|=0$$ (6)

Thus, the level set function can be solved by finding a velocity field *v*_*n*_ to maintain the first-order Hamilton-Jacobi partial differential equation (H-J PDE) in Eq. 6. In the PLSM (Luo et al., 2007), the level set function is interpolated via centrally positioned CSRBFs at a set of given fixed knots *N* over the whole design domain by

$$\Phi \left(x,t\right)=\varphi {\left(x\right)}^{T}\alpha \left(t\right)=\sum_{i=1}^{N}\varphi \left(x\right)\alpha_{i}\left(t\right)\;$$ (7)

The vector with the CSRBFs functions is defined as:

$$\varphi \left(x\right)={\left[\phi_{1}\left(x\right),\;\phi_{2}\left(x\right),\;...\;,\;\phi_{N}\left(x\right)\right]}^{T}$$ (8)

The expansion coefficient vector is given by:

$$\alpha \left(t\right)={\left[\alpha_{1}\left(t\right),\;\alpha_{2}\left(t\right),\;...\;,\;\alpha_{N}\left(t\right)\right]}^{T}$$ (9)

In this paper, the CSRBFs with C2 continuity is used (Luo et al., 2007; Luo et al., 2009). Because of CSRBF knots fixed in space, the shape functions *φ* (*x*) are spatial only and the expansion coefficients *α* (*t*) are time-dependent only, leading to a separation of the time and space in the level set function. Thus, the H-J PDE is transformed as the following ordinary differential equation system:

$$\varphi {\left(X\right)}^{T}\dot{\alpha }\left(t\right)-v_{n}\left|{\left(\nabla \varphi \right)}^{T}\alpha \left(t\right)\right|=0$$ (10)

In this way, the normal velocity field is only determined by the parameters *α* (*t*):

$$v_{n}=\frac{\varphi {\left(X\right)}^{T}}{\left|{\left(\nabla \varphi \right)}^{T}\alpha \left(t\right)\right|}\dot{\alpha }\left(t\right),\text{ where }\dot{\alpha }\left(t\right)=\frac{d\alpha \left(t\right)}{dt}$$ (11)

## Darcy-Stokes Coupling

The application of the Darcy-Stokes coupling approach in saturated porous mediums (Guest and Prévost, 2006) provides an efficient way to deal with the moving-boundary no-slip conditions for solid-fluid interfaces. In the system, the solid region is treated as a porous medium with flow governed by Darcy’s law, and the fluid area is treated as Stokes flow. A unified analysis model that combines the Darcy and Stokes equations can then be established. In the model, a penalization to flow in the solid region is taken by assigning a low permeability to drive the velocity to close zero. Thus, the no-slip conditions along the solid-fluid interface are maintained (Guest and Prévost, 2006; Wang et al., 2016). By using the Darcy-Stokes coupling method, no additional boundary conditions are needed for solid-fluid interfaces, which saves the computational time and benefits the convergence of the optimization.

Stokes flow is usually used to describe a steady viscous fluid with slow velocity by ignoring convective and inertia items in the momentum equations. A typical Stokes flow is formulated in Eq. 4. Darcy flow, derived from the NS equations, usually describes a fluid through a porous medium by the homogenization method (Whitaker, 1986). In this work, the solid regions in the design domain can be treated as porous mediums govern by Darcy flow, where the nodal velocities are close to zero. The typical equations of Darcy flow are given as:

$$\begin{array}{l} u=-\frac{k}{\mu }\left(\nabla p-f\right)\;\;\;\;\;\;\;\text{in}\;{\text{Ω}}_{\text{f}}\\ \nabla \cdot u=0\;\;\;\;\;\;\;\text{in}\;{\text{Ω}}_{\text{f}}\\ u=0\;\;\;\;\;\;\;\text{on}\;{\text{Γ}}_{\text{f}} \end{array}$$ (12)

where *k* is the fluid permeability, and **f** is the vector of external force. When using the Darcy-Stokes method, the velocity and pressure are dependent variables but with different orders in both flows. The incompressible condition has a stability requirement for the combination of the interpolation functions. Here, the stabilized mixed finite element methods (Hughes et al., 1986) is used for Stokes flow, and the method proposed by Masud and Hughes (2002) is applied for Darcy flow. Both stabilized mixed methods can avoid restrictions of the Babuška-Brezzi conditions (Babuška, 1971).

The stabilized matrix form for Darcy-Stokes coupling is given by

$$\left[\begin{array}{cc} K_{\mathrm{ds}} & -G_{ds}\\ G_{\text{ds}}^{\text{T}} & M_{ds} \end{array}\right]\left[\begin{array}{c} u\\ p \end{array}\right]=\left[\begin{array}{c} F_{ds}\\ H_{ds} \end{array}\right]$$ (13)

Combined within the PLSM, the matrices in the Darcy-Stokes system are defined as:

$$\begin{array}{l} K_{\text{ds}}\left(x\right)=A\left\{H\left(\text{Φ}\left(x\right)\right)K_{\text{d}}+\left[1-H\left(\text{Φ}\left(x\right)\right)\right]K_{\text{s}}\right\}\\ G_{\text{ds}}\left(x\right)=A\left\{H\left(\text{Φ}\left(x\right)\right)G_{\text{d}}+\left[1-H\left(\text{Φ}\left(x\right)\right)\right]G_{\text{s}}\right\}\\ G_{\text{ds}}^{\text{T}}\left(x\right)=A\left\{H\left(\text{Φ}\left(x\right)\right)G_{\text{d}}^{\text{T}}+\left[1-H\left(\text{Φ}\left(x\right)\right)\right]\left(L_{\text{s}}+G_{\text{s}}^{\text{T}}\right)\right\}\\ M_{\text{ds}}\left(x\right)=A\left\{H\left(\text{Φ}\left(x\right)\right)M_{\text{d}}+\left[1-H\left(\text{Φ}\left(x\right)\right)\right]M_{\text{s}}\right\} \end{array}$$ (14)

where *A* is the standard finite element routine. The subscript “ds” denotes the matrices defined in the Darcy-Stokes system. The subscripts “d” and “s” emphasize Darcy and Stokes flow, respectively. **u** is the velocity vector, and **p** is the pressure vector. **K** is the viscosity stiffness matrix, **G** is the gradient matrix, **G** ^T^ is the divergence matrix, **L** is the consistency matrix, and **M** is the stabilization matrix. **F** and **H** are the nodal forces. *H* [Φ(*x*)] is the Heaviside function (Wang et al., 2003; Luo et al., 2007) of Φ(*x*) at point *x*. The interpretations indicate solid elements have Darcy stiffness, and void elements have Stokes stiffness.

## Homogenization of the Stent Properties

The stenting structure consists of periodic array of identical microstructures. A near wall horizontal cross-section profile of the microstructure is specified as the 2D design domain. The numerical homogenization method is adopted to compute the effective elasticity tensor and the MFP tensor.

### Homogenization of Elasticity

The effective elasticity tensor *D* ^H^_*ijkl*_ of the microstructure is assessed by the homogenization method as:

$$D_{ijkl}^{\text{H}}=\frac{1}{\left|Y\right|}\int_{Y}\left(\varepsilon_{pq}^{0\left(ij\right)}-\varepsilon_{pq}^{*\left(ij\right)}\right)D_{pqrs}\left(\varepsilon_{pq}^{0\left(kl\right)}-\varepsilon_{pq}^{*\left(kl\right)}\right)dY$$ (15)

where *Y* is the micro design domain and denotes one unit cell of the stent, and |*Y*| is the area of the unit cell. *i*, *j*, *k*, *l* = 1, 2. *D*_*pqrs*_ is the elasticity tensor of the solid material in the design domain. $\varepsilon_{\mathrm{pq}}^{0\mathrm{(ij)}}$ is the test unit strain field, where (1,0,0)^*T*^, (0,1,0)^*T*^ and (0,0,1)^*T*^ are used in 2D models. $\varepsilon_{\mathrm{pq}}^{*\mathrm{(ij)}}$ is the locally varying strain fields and defined by:

$$\varepsilon_{pq}^{*\left(ij\right)}=\frac{1}{2}\left(\frac{\partial u_{p}^{ij}}{\partial y_{q}}+\frac{\partial u_{q}^{ij}}{\partial y_{p}}\right)$$ (16)

The displacement field *u* ^*(ij)*^ is calculated by applying the periodical boundary conditions as

$$\int_{Y}\left(\varepsilon_{pq}^{0\left(ij\right)}-\varepsilon_{pq}^{*\left(ij\right)}\left(u^{\left(ij\right)}\right)\right)D_{pqrs}\varepsilon_{rs}^{*\left(kl\right)}\left(v^{\left(kl\right)}\right)dY=0,\;\;\forall \;v^{\left(kl\right)}\in U_{p}\left(Y\right)$$ (17)

where *ν* ^*(kl)*^ is a virtual displacement field in *U*_*p*_(*Y*). Note that *U* is the kinematically admissible displacement space comprised of periodic Y.

The effective stiffness matrix of the material that has four independent components is defined by

$$D^{\text{H}}=\left[\begin{array}{ccc} D_{11}^{\text{H}} & D_{12}^{\text{H}} & 0\\ D_{12}^{\text{H}} & D_{22}^{\text{H}} & 0\\ 0 & 0 & D_{33}^{\text{H}} \end{array}\right]$$ (18)

The auxetic properties can be obtained through the design having effective negative Poisson’s ratios (NPR). Here, two Poisson’s ratios *Mu1* and *Mu2* in two directions are used to define the related design objectives, and the calculations of the two Poisson’s ratios are given by:

$$\{\begin{array}{c} Mu1=D_{12}^{\text{H}}/D_{11}^{\text{H}}\\ Mu2=D_{12}^{\text{H}}/D_{22}^{\text{H}} \end{array}$$ (19)

### Homogenization of Modified Fluid Permeability

A permeability in fluid mechanics is usually used to quantify the ability of a porous material for fluids to go through it. The permeability can also be applied to measure the obstructions caused by stents. Considering the minimization of the stenting hemodynamic alterations, the optimization aims to reduce the obstruction effect of the stent on the blood flow. Since the blood flow is above the stent layer, rather than across through the stent region, the MFP is different from the typical fluid permeability. By applying the homogenization method in Stokes equations, Darcy’s law can be obtained:

$$U=-\frac{1}{\mu }K^{\text{H}}\left(\nabla p-f\right)$$ (20)

where **U** is the vector of average velocities. *μ* and **f** are the viscosity and the external body force, respectively. ∇p is the pressure gradient. **K** ^H^ is the homogenized effective fluid permeability tensor. The calculation of a typical permeability can be formulated as:

$$K^{\text{H}}=\left[K_{\text{ij}}^{\text{H}}\right]=\frac{1}{\left|Y\right|}{\left(w^{\left(i\right)}\right)}^{T}K_{\text{ds}}w^{\left(j\right)}$$ (21)

where the effective fluid permeability tensor **K** ^**H**^ is assembled by the components *K* ^H^_ij_ in the principal direction. **w** is the velocity vector solved from the above Darcy-Stokes coupling system via Eq. 13. K_ds_ is the Darcy-Stokes viscosity matrix defined in Eq. 14. After that, the MFP tensor is proposed for the 2D design domain. The formulation of the MFP derived from Eq. 21 can be defined as:

$$K_{2\text{D}}^{\text{H}}=\left[K_{2\text{D}\left(ij\right)}^{\text{H}}\right]=\frac{1}{\left|Y_{2D}\right|}{\left(w_{2D}^{\left(i\right)}\right)}^{T}K_{\text{ds}}^{2\text{D}}w_{2D}^{\left(j\right)}$$ (22)

where the subscript “2D” denotes the extracted 2D design domain. **K** ^H^_2D_ is the MFP tensor. **w**_2D_ is the velocity vector in the 2D design domain, which is calculated in the 3D computational domain. **K** ^2D^_ds_ is the Darcy-Stokes viscosity matrix defined for the 2D design domain and derived from the mixed viscosity matrix **K**_ds_. The velocity vector **w**_2D_ contains two directional components: one is in the blood flow direction, and another one is normal to the blood flow. Thus, the purpose of reducing the stenting obstructions can be obtained by minimizing the vertical component of the MFP.

## Optimization Formulation and Sensitivity Analysis

This section will formulate the multiscale multi-objective topology optimization problem, and the sensitivity analyse of the optimization objectives will also be derived. Since different objectives will be associated with different design variables. The mechanical objectives are auxetic properties and stent stiffness, while the MFP is related to CFD simulation. After that, the connection between the MFP and design variables can be built, and the sensitivity analysis can be performed.

### Optimization Model

The numerical optimization starts from an initial guess in the 2D design domain, where the expansion coefficients of the CS-RBF interpolation in the PLSM are defined as design variables. Then, the three objectives are computed simultaneously. With respect to the mechanical objectives, the compliance of the macroscopic stenting structure can be computed by the obtained effective elasticity tensor. Regarding the fluidic objective, the 3D computational domain is first established based on the initial 2D design domain. The material distribution of the 3D microstructure is determined by the 2D profile in the design domain. The surrounding blood regions are specified the same as the initial definition in Figure 1. The coupled Darcy-Stokes system can be solved, and the velocity vector of the 3D fluid model is then achieved. After that, the velocity vector of the 2D design domain is extracted from the 3D fluid model to calculate the MFP. Thus, all three objectives and related sensitivities are obtained. They are then normalized and weighted as an equivalent objective function. Based on the sensitivity information, the PLSM is utilized to update the structural topological shapes.

The proposed multiscale topology optimization model is formulated as:

$$\left\{ \begin{array}{l} F\;i\;n\;d\;\;\;\;\alpha_{2D,n}^{MI}\left(n=1,\;2,\;...\;,\;N\right)\\ M\;i\;n\;\;\;\;\;J\left(\alpha_{2D}^{MI}\right)=W_{1}J_{1}\left(\alpha_{2D}^{MI}\right)+W_{2}J_{2}\left(\alpha_{2D}^{MI}\right)+W_{3}J_{3}\left(\alpha_{2D}^{MI}\right)\\ \;=W_{1}J_{A}^{MI}\left(\alpha_{2D}^{MI}\right)+W_{2}J_{P}^{MI}\left(\alpha_{2D}^{MI}\right)+W_{3}J^{MA}\left(\alpha_{2D}^{MI}\right)\\ S.\;\;t.\;\;\;\;\;\;\;V\left(\alpha_{2D}^{MI}\right)=\int_{\Omega^{MI}}H\left(\Phi^{MI}\left(\alpha_{2D}^{MI}\right)\right)d\Omega_{2D}^{MI}-V_{2D}^{max}\leq 0\\ \;\;\;\;\;\;\;\;\;\;\;F_{2D}^{MI}\left(u_{2D}^{MI},w_{2D}^{MI},\alpha_{2D}^{MI}\right)=L_{2D}^{MI}\left(w_{2D}^{MI},\;\alpha_{2D}^{MI}\right),\;\forall \;w_{2D}^{MI}\in \bar{U}\left(\Omega_{2D}^{MI}\right)\\ \;\;\;\;\;\;\;\;\;\;\;F_{3D}^{MI}\left(u_{3D}^{MI},p_{3D}^{MI},v_{3D}^{MI},q_{3D}^{MI},\alpha_{2D}^{MI}\right)=L_{3D}^{MI}\left(v_{3D}^{MI},q_{3D}^{MI}\right),\;\forall \;v_{3D}^{MI},q_{3D}^{MI}\in \bar{U}\left(\Omega_{3D}^{MI}\right)\\ \;\;\;\;\;\;\;\;\;\;\;F_{2D}^{MA}\left(u_{2D}^{MA},w_{2D}^{MA},D_{ijkl}^{H}\right)=L_{2D}^{MA}\left(w_{2D}^{MA}\right),\;\forall \;w_{2D}^{MA}\in \bar{U}\left(\Omega_{2D}^{MA}\right)\\ \;\;\;\;\;\;\;\;\;\;\;\alpha_{2D,min}^{MI}\leq \alpha_{2D,n}^{MI}\leq \alpha_{2D,max}^{MI} \end{array}\right.$$ (23)

where $J_{A}^{MI}={\left(Mu1\left(\alpha_{2D}^{MI}\right)+1\right)}^{2}+{\left(Mu2\left(\alpha_{2D}^{MI}\right)+1\right)}^{2}$, $J_{P}^{MI}=K_{2\text{D}}^{\text{H}\left(2,2\right)}\left(\alpha_{2D}^{MI}\right)$ and $J^{MA}=\frac{1}{2}\int_{\Omega_{2D}^{MA}}\varepsilon_{ij}^{T}\left(u_{2D}^{MA}\right)D_{ijkl}^{H}\left(\alpha_{2D}^{MI}\right)\varepsilon_{kl}\left(u_{2D}^{MA}\right)d\Omega_{2D}^{MA}$.

*J* is the objective function, consisting of the auxetic property $J_{A}^{MI}$, the fluid objective $J_{p}^{MI}$, and macro compliance *J* ^*MA*^, where *W*_*1*_, *W*_*2*_, *W*_*3*_ are corresponding weight factors. The superscript “*MA*” and “*MI*” denote parameters in macroscale and microscale, respectively; the subscript “*2D*” refers to parameters in the 2D design domain, and “*3D*” means parameters in 3D domain. *N* is the total number of fixed knots in the 2D design domain. The coefficients of the interpolation $\alpha_{2D,n}^{MI}$ are the design variables, ranging from $\alpha_{2D,\min}^{MI}$ to $\alpha_{2D,\max}^{MI}$. The subscript “*A*” and “*P*” denote auxetic and permeability, respectively. **K** ^H^_2D_ is the MFP, and **K** ^H(2,2)^_2D_ is the vertical component. *V* is the volume constraint with the upper limit $V_{\mathrm{2D}}^{Max}$, applied to the 2D microstructure but can also restrict the 3D microstructure.

The MFP related to the design variables can be calculated by:

$$K_{2\text{D}}^{\text{H}}\left(\alpha_{2D}^{MI}\right)=\left[K_{2D\left(ij\right)}^{\text{H}}\left(\alpha_{2D}^{MI}\right)\right]=\frac{1}{\left|\Omega_{2D}^{MI}\right|}{\left(w_{2D}^{\left(i\right)}\right)}^{T}K_{\text{ds}}^{2\text{D}}\left(H^{f}\left(\Phi_{2D}^{MI}\left(\alpha_{2D}^{MI}\right)\right)\right)w_{2D}^{\left(j\right)}$$ (24)

where the velocity vector **w**_*2D*_ is extracted from the velocity vector $u_{\mathrm{3D}}^{MI}u_{\mathrm{3D}}^{MI}$, which is calculated by the following momentum equation performed in the 3D computational domain.

$$F_{3D}^{MI}\left(u_{3D}^{MI},p_{3D}^{MI},v_{3D}^{MI},q_{3D}^{MI},\alpha_{2D}^{MI}\right)=L_{3D}^{MI}\left(v_{3D}^{MI},q_{3D}^{MI}\right)$$ (25)

where $v_{3D}^{MI}$ and $P_{3D}^{MI}$ are the boundary conditions defined in the CFD model, given by

$$\left[\begin{array}{cc} K_{ds}\left(H^{f}\right) & -G_{ds}\left(H^{f}\right)\\ G_{ds}^{T}\left(H^{f}\right) & M_{ds}\left(H^{f}\right) \end{array}\right]\left[\begin{array}{c} u\left(u_{3D}^{MI}\right)\\ p\left(p_{3D}^{MI}\right) \end{array}\right]=\left[\begin{array}{c} F_{ds}\left(w_{3D}^{MI}\right)\\ H_{ds}\left(q_{3D}^{MI}\right) \end{array}\right]$$ (26)

where *H* ^*f*^ denotes the Heaviside function *H* applied to the Darcy-Stokes coupling in the CFD model. *Θ* in *H* ^*f*^ is equal to zero to identify fluid material in the design domain. The $D_{ijkl}^{H}$ used for the evaluation of the mechanical properties in the optimization model can be calculated by:

$$D_{ijkl}^{\text{H}}\left(\alpha_{2D}^{MI}\right)=\frac{1}{\left|\Omega_{2D}^{MI}\right|}\int_{\Omega_{2D}^{MI}}\left(\varepsilon_{pq}^{0\left(ij\right)}-\varepsilon_{pq}^{*}\left(u_{2D}^{\left(ij\right)}\right)\right)D_{pqrs}\left(\varepsilon_{rs}^{0\left(kl\right)}-\varepsilon_{rs}^{*}\left(u_{2D}^{\left(kl\right)}\right)\right)H\left(\Phi_{2D}^{MI}\left(\alpha_{2D}^{MI}\right)\right)d\Omega_{2D}^{MI}$$ (27)

where *D*_*pqrs*_ is the elasticity tensor of the solid. $\varepsilon_{\mathrm{pq}}^{0}$ is the test unit strain field, where (1,0,0)^*T*^, (0,1,0)^*T*^ and (0,0,1)^*T*^ are used. $\varepsilon_{\mathrm{pq}}^{*}$ is the strain field related to the displacement *u*_*2D*_ that can be calculated by

$$\int_{\Omega_{2D}^{MI}}\left(\varepsilon_{pq}^{0\left(ij\right)}-\varepsilon_{pq}^{*}\left(u_{2D}^{MI\left(ij\right)}\right)\right)D_{pqrs}\varepsilon_{rs}^{*}\left(w_{2D}^{MI\left(kl\right)}\right)H\left(\Phi_{2D}^{MI}\right)d\Omega_{2D}^{MI}=0,\;\;\forall \;w\in \bar{U}\left(\Omega_{2D}^{MI}\right)$$ (28)

where *w* is the virtual displacement field.

The bilinear energy and the linear load forms in the 2D microscale domain are given by

$$F_{2D}^{MI}\left(u_{2D}^{MI},w_{2D}^{MI},\alpha_{2D}^{MI}\right)=\int_{\Omega_{2D}^{MI}}\varepsilon_{ij}^{*}\left(u_{2D}\right)D_{pqrs}\varepsilon_{kl}^{*}\left(w_{2D}^{MI}\right)H\left(\Phi_{2D}^{MI}\left(\alpha_{2D}^{MI}\right)\right)d\Omega_{2D}^{MI}$$ (29)

$$L_{2D}^{MI}\left(w_{2D}^{MI},\;\alpha_{2D}^{MI}\right)=\int_{\Omega_{2D}^{MI}}\varepsilon_{ij}^{0\left(ij\right)}\left(u_{2D}\right)D_{pqrs}\varepsilon_{kl}^{*}\left(w_{2D}^{MI}\right)H\left(\Phi_{2D}^{MI}\left(\alpha_{2D}^{MI}\right)\right)d\Omega_{2D}^{MI}$$ (30)

The bilinear energy and the linear load forms in the 2D macroscale domain can be computed by:

$$F_{2D}^{MA}\left(u_{2D}^{MA},w_{2D}^{MA},D_{ijkl}^{H}\right)=\int_{\Omega_{2D}^{MA}}\varepsilon_{ij}\left(u_{2D}^{MA}\right)D_{ijkl}^{H}\varepsilon_{kl}\left(w_{2D}^{MA}\right)d\Omega_{2D}^{MA}$$ (31)

$$L_{2D}^{MA}\left(w_{2D}^{MA}\right)=\int_{\Omega_{2D}^{MA}}pw_{2D}^{MA}d\Omega_{2D}^{MA}+\int_{\Omega_{2D}^{MA}}\tau w_{2D}^{MA}d\Gamma_{2D}^{MA}$$ (32)

where *p* is the body force, and *τ* is the traction of the boundary $\Gamma_{\mathrm{2D}}^{MA}$ in the 2D macroscale.

### Sensitivity Analysis

Based on the topology optimization formulation, the sensitivities can be obtained via the first-order derivatives of the objective functions with respect to the design variables $\alpha_{2D}^{MI}$.

The sensitivity of the macroscopic compliance is computed by:

$$\frac{\partial J^{MA}}{\partial \alpha_{2D}^{MI}}=\frac{1}{2}\int_{\Omega_{2D}^{MA}}\varepsilon_{ij}^{T}\left(u_{2D}^{MA}\right)\frac{\partial D_{ijkl}^{H}\left(\alpha_{2D}^{MI}\right)}{\partial \alpha_{\xi }^{M\;I}}\varepsilon_{kl}\left(u_{2D}^{MA}\right)d\Omega_{2D}^{MA}$$ (33)

where the first-order derivatives of the effective elasticity tensor $D_{ijkl}^{H}$ with respect to t is:

$$\frac{\partial D_{ijkl}^{H}}{\partial t}=\frac{1}{\left|\Omega_{2D}^{MI}\right|}\int_{\Omega_{2D}^{MI}}\beta_{2D}^{MI}v^{n}\left|{\left(\nabla \Phi_{2D}^{MI}\right)}^{T}\right|\delta \left(\Phi_{2D}^{MI}\right)d\Omega_{2D}^{MI}$$ (34)

$$\beta_{2D}^{MI}=\left(\varepsilon_{pq}^{0\left(ij\right)}-\varepsilon_{pq}^{*}\left(u_{2D}^{MI\left(ij\right)}\right)\right)D_{pqrs}\left(\varepsilon_{rs}^{0\left(kl\right)}-\varepsilon_{rs}^{*}\left(u_{2D}^{MI\left(kl\right)}\right)\right)$$ (35)

where *v* ^*n*^ is determined in Eq. 11 and is then substituted into Eq. 34:

$$\frac{\partial D_{ijkl}^{H}}{\partial t}=\left(\frac{1}{\left|\Omega_{2D}^{MI}\right|}\int_{\Omega_{2D}^{MI}}\beta_{2D}^{MI}\varphi_{2D}^{MI}{\left(x\right)}^{T}\delta \left(\Phi_{2D}^{MI}\right)d\Omega_{2D}^{MI}\right){\dot{\alpha }}_{2D,n}^{MI}\left(t\right)$$ (36)

In terms of the chain rule, the first-order derivatives of DH ijkl with respect to t can also be given as:

$$\frac{\partial D_{ijkl}^{H}}{\partial t}=\frac{\partial D_{ijkl}^{H}}{\partial \alpha_{2D}^{MI}}{\dot{\alpha }}_{2D,n}^{MI}\left(t\right)$$ (37)

Comparing Eq. 36 and Eq. 37, the first-order derivatives of $D_{ijkl}^{H}$ with respect to the expansion coefficients $\alpha_{2D}^{MI}$ can then be obtained as:

$$\frac{\partial D_{ijkl}^{H}}{\partial \alpha_{2D}^{MI}}=\frac{1}{\left|\Omega_{2D}^{MI}\right|}\int_{\Omega_{2D}^{MI}}\beta_{2D}^{MI}\varphi_{2D}^{MI}{\left(x\right)}^{T}\delta \left(\Phi_{2D}^{MI}\right)d\Omega_{2D}^{MI}$$ (38)

Therefore, the sensitivity of the objective *J* ^*MA*^ can be calculated by substituting Eq. 38 into Eq. 33.

Since the sensitivity of the objective *JMI A* is also based on Eq. 38, it can be calculated by:

$$\frac{\partial J_{A}^{MI}}{\partial \alpha_{2D}^{MI}}=\frac{\partial {\left(Mu1+1\right)}^{2}}{\partial \alpha_{2D}^{MI}}+\frac{\partial {\left(Mu2+1\right)}^{2}}{\partial \alpha_{2D}^{MI}}=\frac{\partial {\left(D_{12}^{H}/D_{11}^{H}+1\right)}^{2}}{\partial \alpha_{2D}^{MI}}+\frac{\partial {\left(D_{12}^{H}/D_{22}^{H}+1\right)}^{2}}{\partial \alpha_{2D}^{MI}}$$ (39)

The sensitivity of the MFP can be derived from the Darcy-Stokes coupling system, so the first-order derivatives of $J_{P}^{MI}$ with respect to the expansion coefficients $\alpha_{2D}^{MI}$ can be given by:

$$\frac{\partial J_{P}^{MI}}{\partial \alpha_{2D}^{MI}}=\frac{\partial K_{2\text{D}}^{\text{H}\left(2,2\right)}\left(\alpha_{2D}^{MI}\right)}{\partial \alpha_{2D}^{MI}}$$ (40)

where the sensitivity of K can be further obtained according to the first order of the Heaviside function *H* ^*f*^ (Dirac function) (Luo, et al., 2007) in the fluid-solid coupling system by

$$\frac{\partial K_{2\text{D}}^{\text{H}}\left(\alpha_{2D}^{MI}\right)}{\partial \alpha_{2D}^{MI}}\;=\frac{1}{\left|\Omega_{2D}^{MI}\right|}\int_{\Omega_{2D}^{MI}}{\left(w_{2D}^{\left(i\right)}\right)}^{T}\varphi_{2D}^{MI}{\left(x\right)}^{T}\delta^{f}\left(\Phi_{2D}^{MI}\right)\left(K_{\text{d}}^{2\text{D}}-K_{\text{s}}^{2\text{D}}\right)w_{2D}^{\left(j\right)}d\Omega_{2D}^{MI}$$ (41)

As discussed previously, the volume constraint is only defined in the 2D design domain but can control the volume of the 3D model. Thus, the sensitivity of the volume constraint can be calculated by:

$$\frac{\partial V}{\partial \alpha_{2D}^{MI}}=\int_{\Omega_{\xi }^{M\;I}}\varphi_{2D}^{MI}{\left(x\right)}^{T}\delta \left(\Phi_{2D}^{MI}\left(\alpha_{2D}^{MI}\right)\right)d\Omega_{2D}^{MI}$$ (42)

After that, the non-dimensional sensitivities for the three objective functions can be given by Eq. 43, which benefits the multi-objective optimization (Marler and Arora, 2004).

$$\frac{\partial J_{i}^{Non}}{\partial \alpha }=\frac{\partial J_{i}/\partial \alpha }{\left|\partial J_{i}^{\max}/\partial \alpha \right|}\;\;\;\;i\in \left(1,2,3\right)$$ (43)

where *i* denotes the three individual objective functions.

## Numerical Results

**FIGURE 4:**
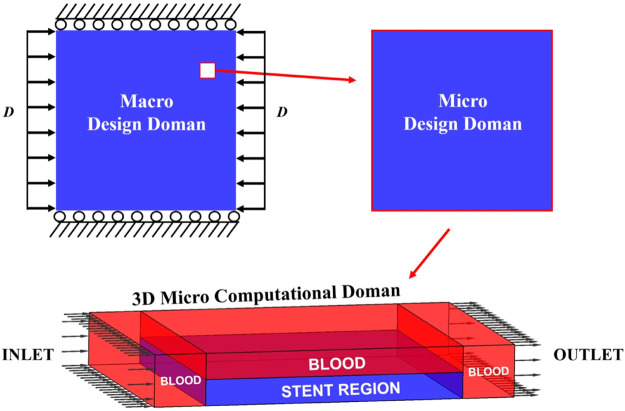
Multi-domain of the numerical model.

The numerical optimization is implemented via MATLAB 2018b to obtain the stenting microstructures. The multi-domain involved in the optimization model is displayed in Figure 4. From the figure, we can see three domains are defined in the model. The stent is deployed as a 2D structure, and it is the macro design domain that consists of 20 × 20 uniform microstructures. The macroscopic boundary and loads conditions: the vertical degree of freedoms of the top and bottom edges are fixed, and horizontal unit displacements are applied on the left and right edges. The stenting microstructures is then defined as the micro design domain. The 2D micro design domain can be treated as the constant horizontal cross-section profile of a 3D microstructure, which is utilized to establish a 3D micro computational domain for the related CFD analysis. In the 3D field, a scaled velocity of blood flow is specified as the inlet condition, while the zero-pressure boundary is defined as the outlet condition. By considering the computational efficiency, a square element with four nodes is used for the 2D micro and macro design domains; an 8-node brick element is used in the 3D domain.

The multi-objective optimization is formulated by weighting three single objective functions. Different combinations of weight factors can lead to different results. All the three optimization objectives are critical to the design. Compared with the macroscopic compliance, the other two objectives determine the material layout to have auxetic and MFP properties, so larger weighting factors are specified for these two objective functions. Thus, three weight factors, 0.35, 0.35 and 0.30, are used.

The volume fraction of the microstructure in the optimization is another factor in determining the optimization results. It should not be too big to block blood flow, or too small to support the artery. Thus, the volume fractions from 25 to 60% are used to investigate the optimization results. The optimized results are summarized in Table 1, which includes the microstructure of each case, the related 3 × 3 microstructural array, and the blood flow streamlines in the design domain. In the table, the black colour denotes stenting microstructures, and the red colour shows blood.

**TABLE 1:** The results of various volume fractions.

Volume(V)	Microstructure	Microstructure (3 × 3)	Streamline
60% (case 1)	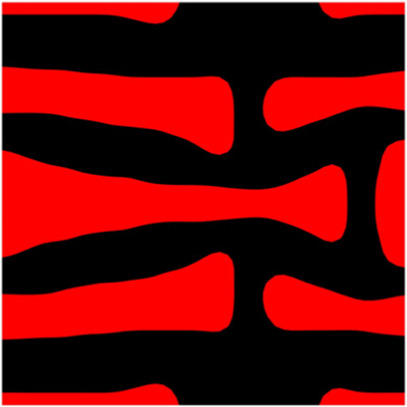	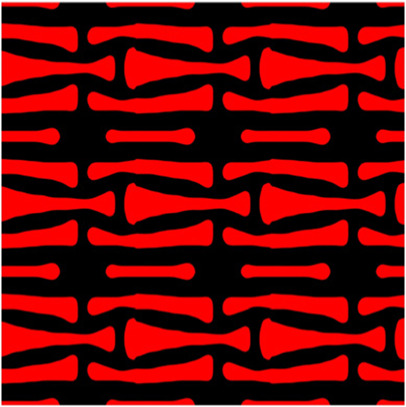	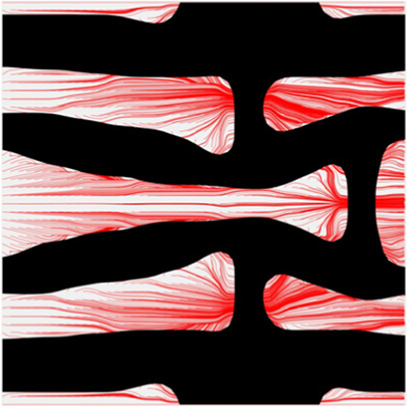
55% (case 2)	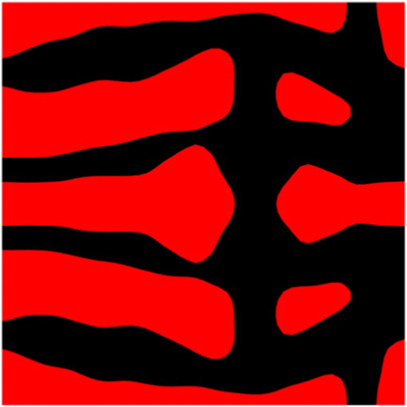	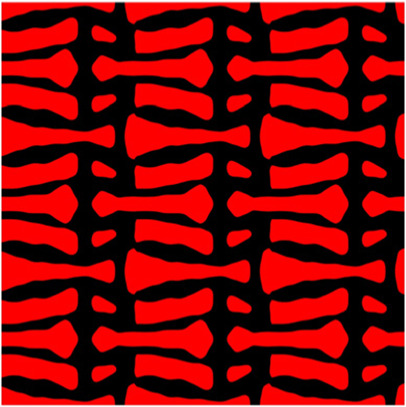	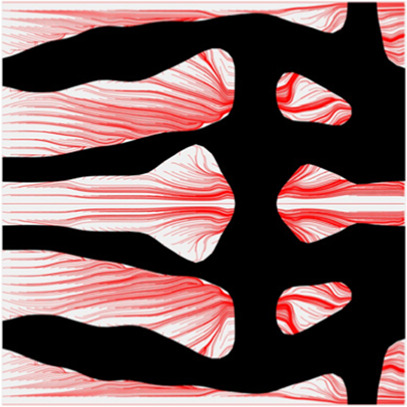
50% (case 3)	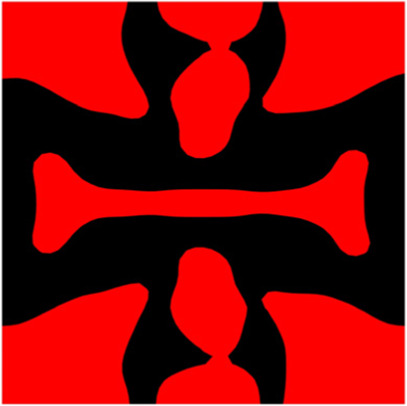	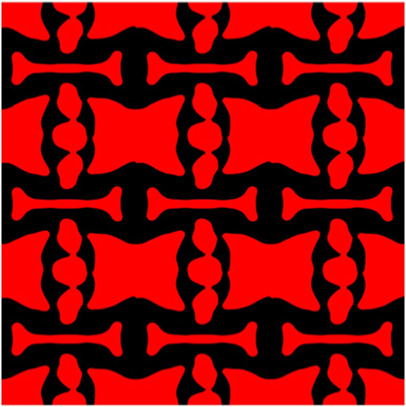	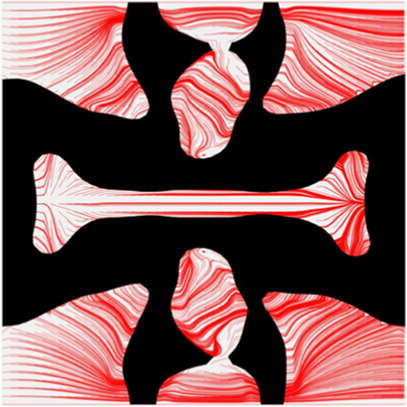
45% (case 4)	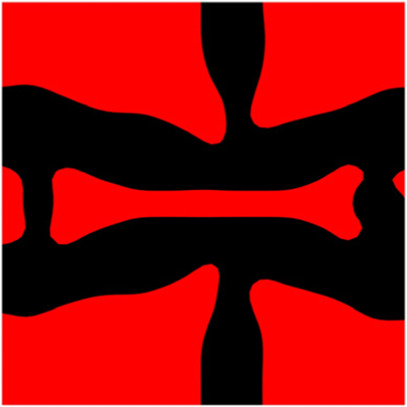	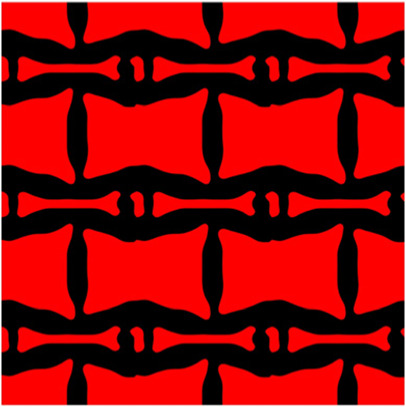	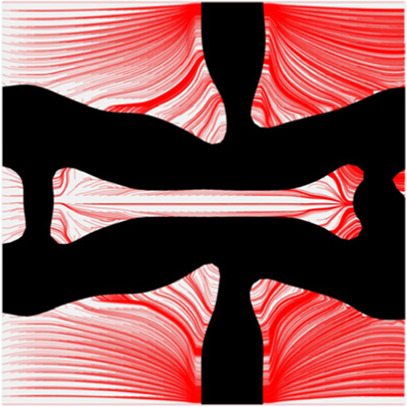
40% (case 5)	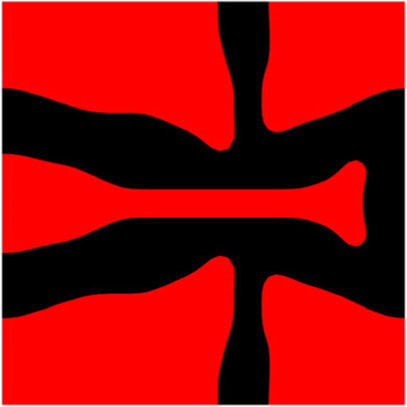	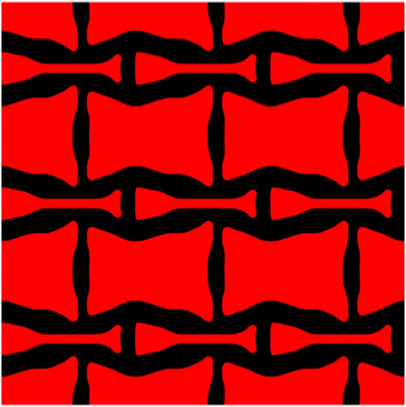	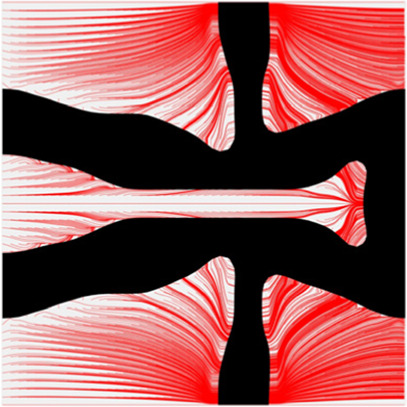
35% (case 6)	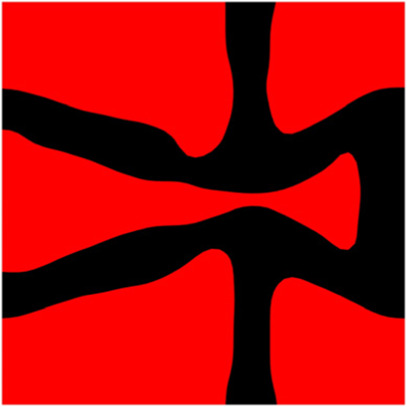	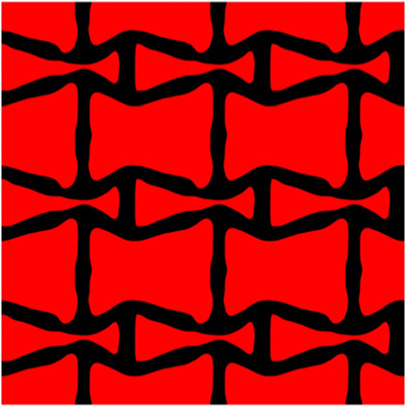	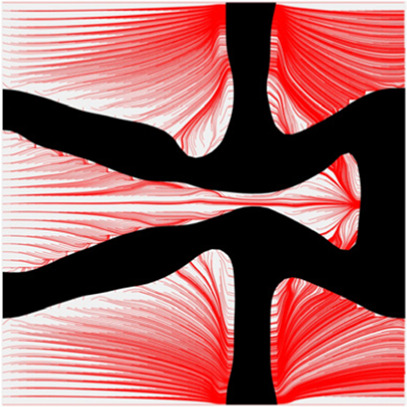
30% (case 7)	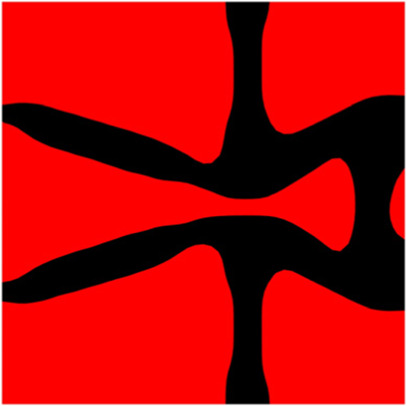	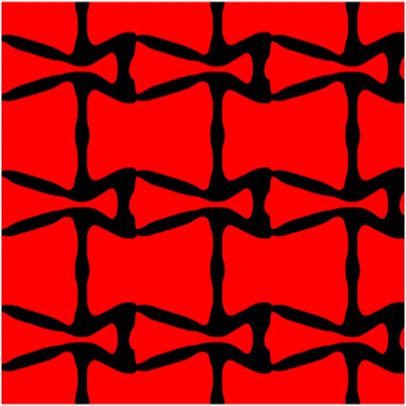	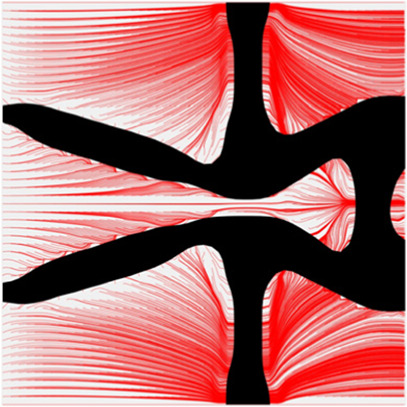
25% (case 8)	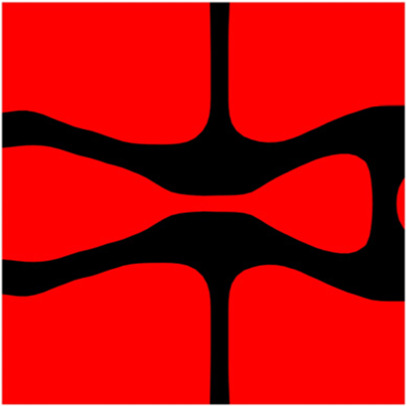	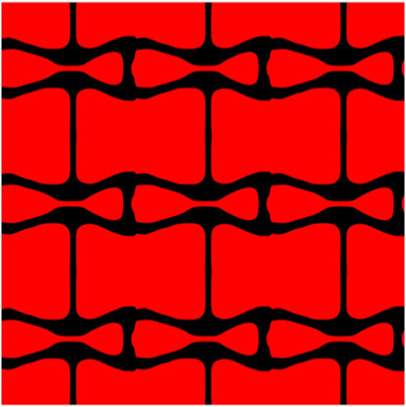	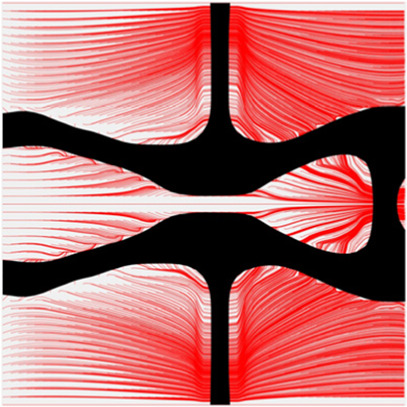

All results in Table 1 show auxetic characteristics: re-entrant connections between struts. The solid material in all designs concentrate more along the direction of the blood flow, which is beneficial for reducing the stenting obstructions. To distinguish different struts in the stenting microstructures, the struts along the blood flow are named horizontal struts, while the others are called vertical struts. When the designs have relatively large volume fractions, such as the cases 1–4, more solid material will be assigned to the horizontal struts to facilitate the blood flow, leading to less or thin vertical struts. When volume fractions are reduced, such as the cases 5–7, the material distributions become more uniform. In these cases, the two horizontal struts are connected by only one vertical strut location on the right edge, exhibiting an opening at the upstream side. This can lead to a long space for blood through and facilitate recovering the disturbed flow. Besides that, the other two vertical struts are also near the right edge to facilitate blood flow. As the volume fraction was further reduced in case 8, the two vertical struts near the horizontal struts become thinner. They are used to connect the stenting unit cells in the same circumference, and two weak connections may lead to stenting fractures. Within the results, case 7 with a 30% volume fraction has more material distribution and few obstructions on the blood flow.

The homogenized effective elasticity tensor and the MFP are given by

$$D^{H}=\left[\begin{array}{ccc} \text{0}\text{.}1082 & -\text{0}\text{.}0339 & 0\\ -\text{0}\text{.}0339 & \text{0}\text{.}0324 & 0\\ 0 & 0 & \text{0}\text{.}0022 \end{array}\right]\;\;\;\;\;\;\;\;\;\;\;\text{where},\;\;\{\begin{array}{c} Mu1=-0.3133\\ Mu2=-1.0463 \end{array}$$ (44)

and

$$K_{2\text{D}}^{\text{H}}=\left[\begin{array}{cc} 1\text{.}0593\times 10^{-3} & 0\\ 0 & 2\text{.}5233\times 10^{-4} \end{array}\right]$$ (45)

It can be found that the values of NPRs are −0.3133 along the flow direction and −1.0463 in the vertical direction, which indicates the microstructure has a relatively smaller vertical stiffness. It means the stenting radial stiffness is smaller than the axial stiffness by adopting the optimized microstructure. The less radial stiffness of the stent can enhance the adaptive deformation and lead to better flexibility. On the other hand, both directions present the auxetic properties that can obtain a smaller volume after compression, so as to facilitate deliverability. The effective MFP can be found in Eq. 45. With respect to the MFP, the design reduces the vertical blood flow which can also leads to directional differences. Hence, the directional differences in both NPR and MFP improve the stenting performances.

**FIGURE 5:**
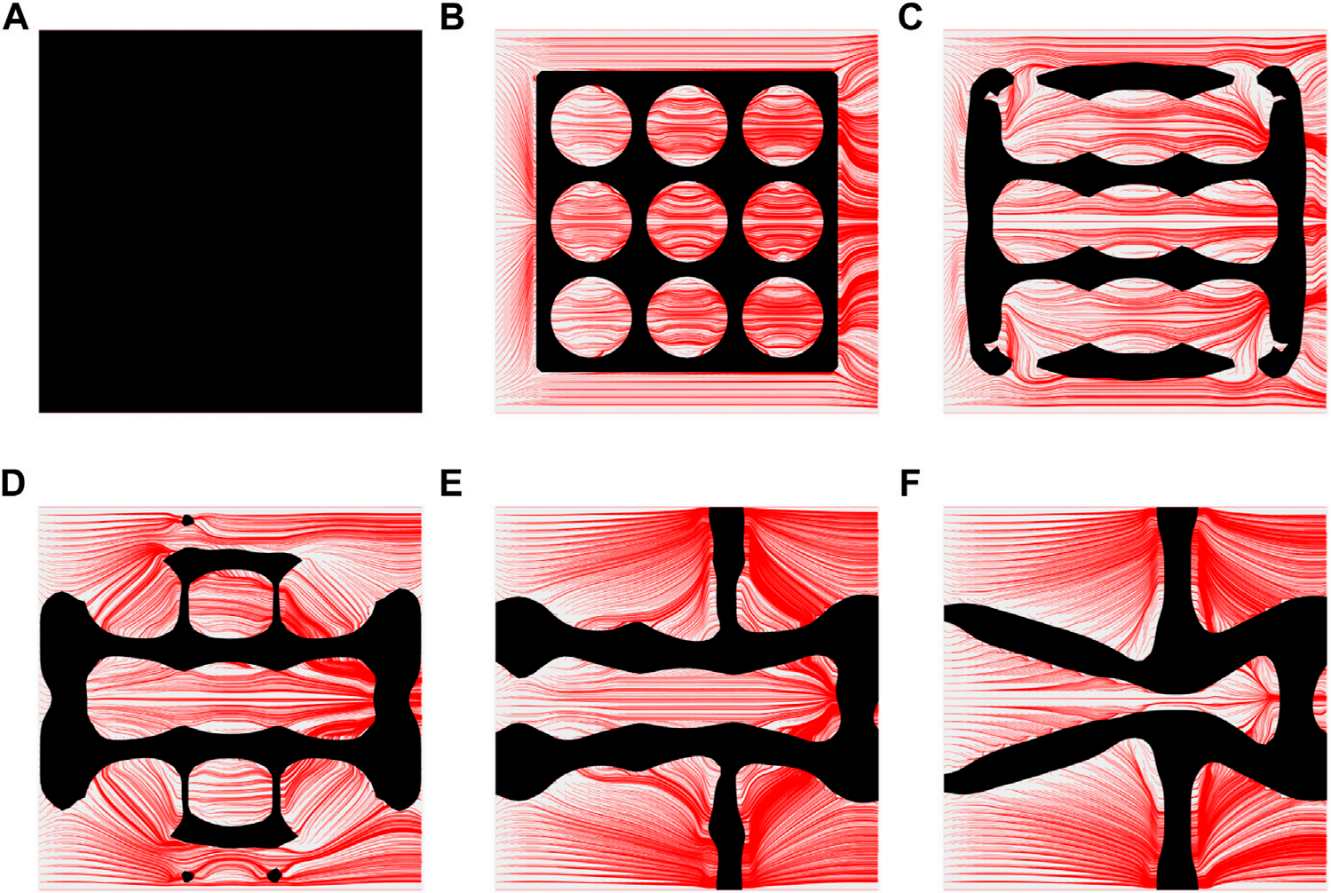
The results of case 7: **(A)** Initial design; **(B–E)** Four intermediate results; **(F)** Final design.

**FIGURE 6:**
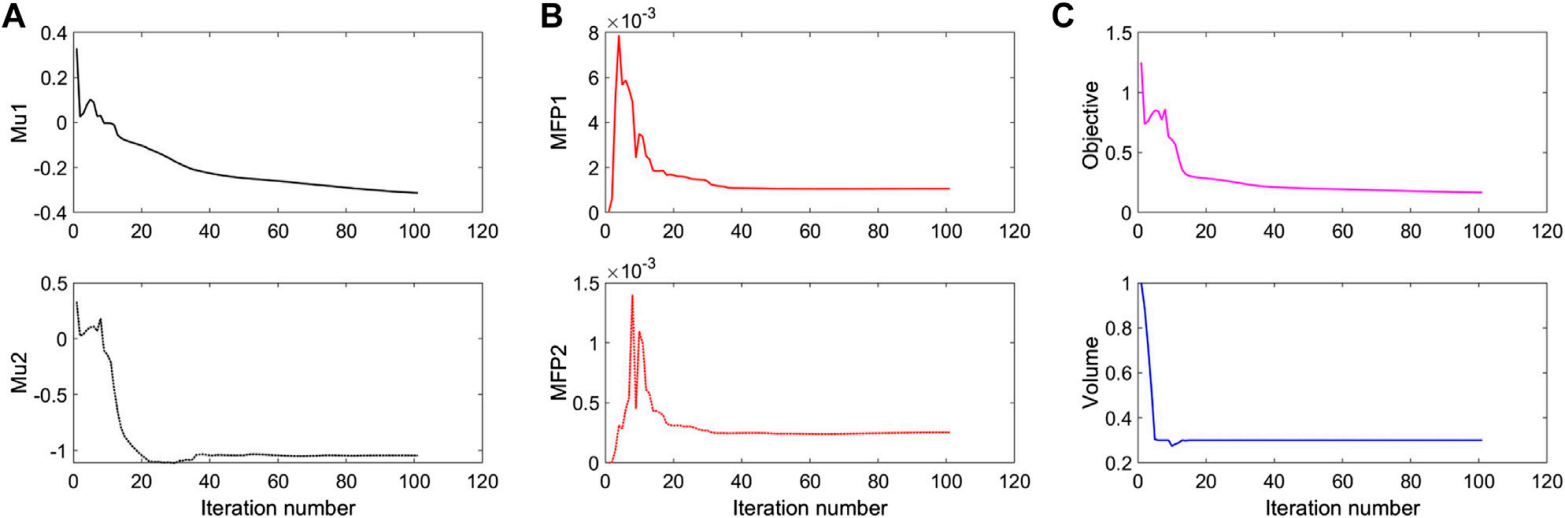
The convergent history of case 7: **(A)** Poisson’s ratios; **(B)** Modified permeabilities; **(C)** Objective and volume fraction.

To further investigate numerical case 7, the dynamic evolutions of the microstructure are presented in Figure 5. We can see the solid material is gradually assigned to the horizontal struts in the optimization process, leading to fewer obstructions to the blood flow. Re-entrant characteristics exhibiting auxetic behaviour can also be created, simultaneously. As shown in Figure 5F, the blood flow is mainly disturbed by the vertical struts, but the normal flow patterns can be recovered behind the struts due to large fluid spaces. Thus, local recirculation regions may occur around the vertical struts but will be small. The convergences of the related objective functions in the optimization are shown in Figure 6. All effective properties, objective functions, and volume constraints are gradually converged to their solutions. We can see that the two MFPs increase from zero to the highest value at the beginning of the optimization, because the solid material fills the design domain at the initial stage. In a word, the optimization process of the case 7 also shows the effectiveness of the optimization.

## Numerical Validations

This section is to numerically validate the performances of the optimized stent. For instance, the minimization of the stent induced adverse hemodynamic can be obtained by reducing the obstructions of the stent to the blood flow. WSS and related derivatives are usually used as the widely accepted metric to measure the stented hemodynamics. Thus, the optimized microstructure is simulated in ANSYS 2019R3 to validate the macroscopic performances. For the optimized stent, the auxetic properties, disturbances to the blood flow, and WSS distributions are numerical validated, respectively.

### Auxetics

**FIGURE 7:**
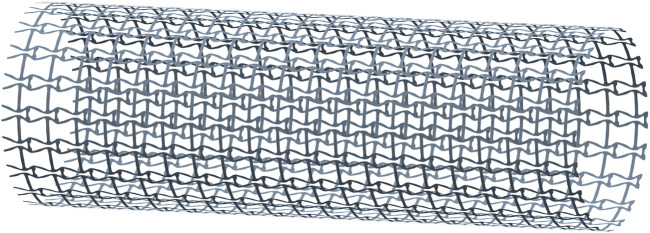
The geometry of the optimized stent.

The auxetic property is one of the design objectives for the stent in the proposed design. Due to the directional differences of the stenting properties, two compression tests are performed: one is under a radial compression load, and the other is under an axial compression load. To create the stenting geometry for the tests, the optimized microstructures are first obtained from MATALB via the STL format, and then ANSYS SpaceClaim module is utilized to finish the creation of the geometry (Figure 7). The stent thickness is 0.1 mm, and the length and the diameter are 12 and 4 mm. In the model, 20 cells are configured along the peripheral direction and 20 cells are arranged along the axial direction.

**FIGURE 8:**
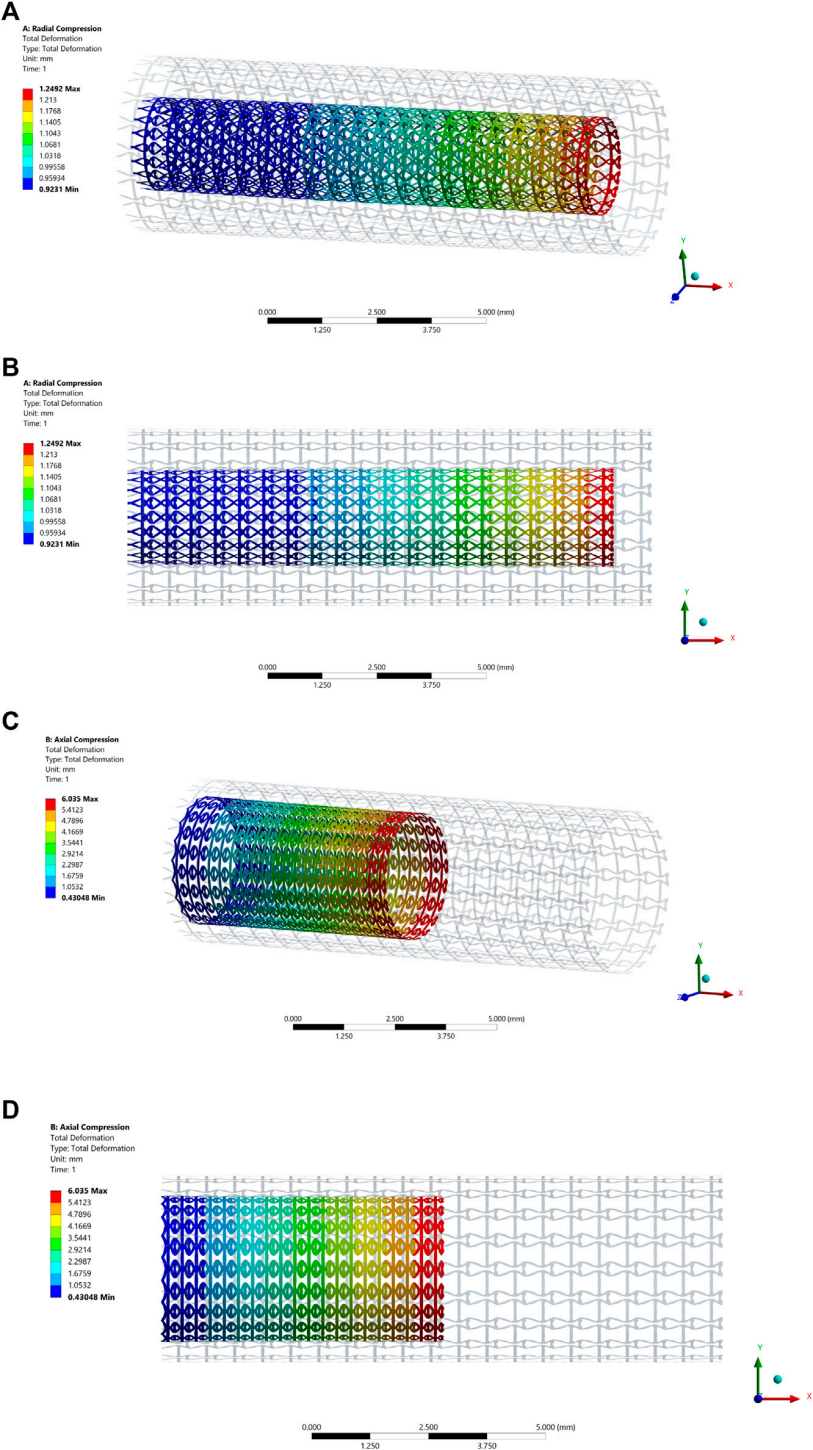
The deformation results of the compression test: **(A)** Axonometric view of the radial compression test result; **(B)** Side view of the radial compression test result; **(C)** Axonometric view of the axial compression test result; **(D)** Side view of the axial compression test result.

The deformation of the radial compression test is shown in Figures 8A,B, where both axonometric and side views are utilized to show its deformation behaviours. A pressure load is applied on the stenting surface to compress the diameter about 50% in the test. From the figure, we can see the stent contracts slightly in the axial direction due to larger axial stiffness. When the stent is compressed in the radial direction to adapt to the various artery shapes, the stenting axial length is relatively stable. It can keep enough length of the stent to cover the lesion even though the stenting radial dimension is changed. The axial compression test result is presented in Figures 8C,D. The test result shows that the stent contracts significantly in the radial direction when applying compression loads in the axial direction from the right side, indicating the NPR behaviour that can improve deliverability.

### Fluid Validations

The disturbance of stents on blood flow is another essential issue in the design. Thus, the simulation for blood flow in the stented segment is performed via the software CFX in ANSYS v2019R3. This research intends to reduce the adverse hemodynamic changes by minimizing the stenting obstructions on the blood flow. In the validations, the streamlines generated by the blood flow are used to investigate the stenting obstructions. WSS distribution is also computed in the simulation.

**FIGURE 9:**
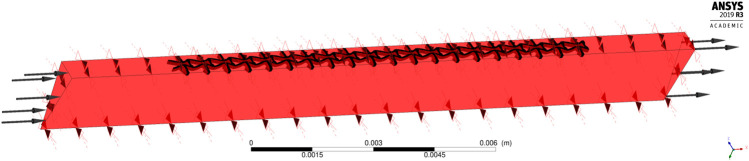
The CFD model.

To save computational cost, the CFD model is established based on 1 cell along the peripheral direction while 20 cells in the axial direction, as shown in Figure 9, where the black one is the stent, and the red is blood. The stent thickness is 0.1 mm, and the length and the diameter are 12 and 4 mm, respectively. By considering the impact of the boundary conditions on flow, the distance between the inlet and the stent struts is specified as 3 mm and another 3 mm between the outlet and the stent. The blood in the model is defined as an incompressible Newtonian viscous fluid. The density is 1,050 kg/m3, and the dynamic viscosity is 3.5 mPa⋅s. The flow is assumed to be steady state with 0.35 m/s inflow velocity and 0 Pa outlet boundary condition. No-slip conditions are applied on the fluid-solid interfaces and the fluid walls. The cylindrical symmetric condition is defined in the peripheral direction.

**FIGURE 10:**
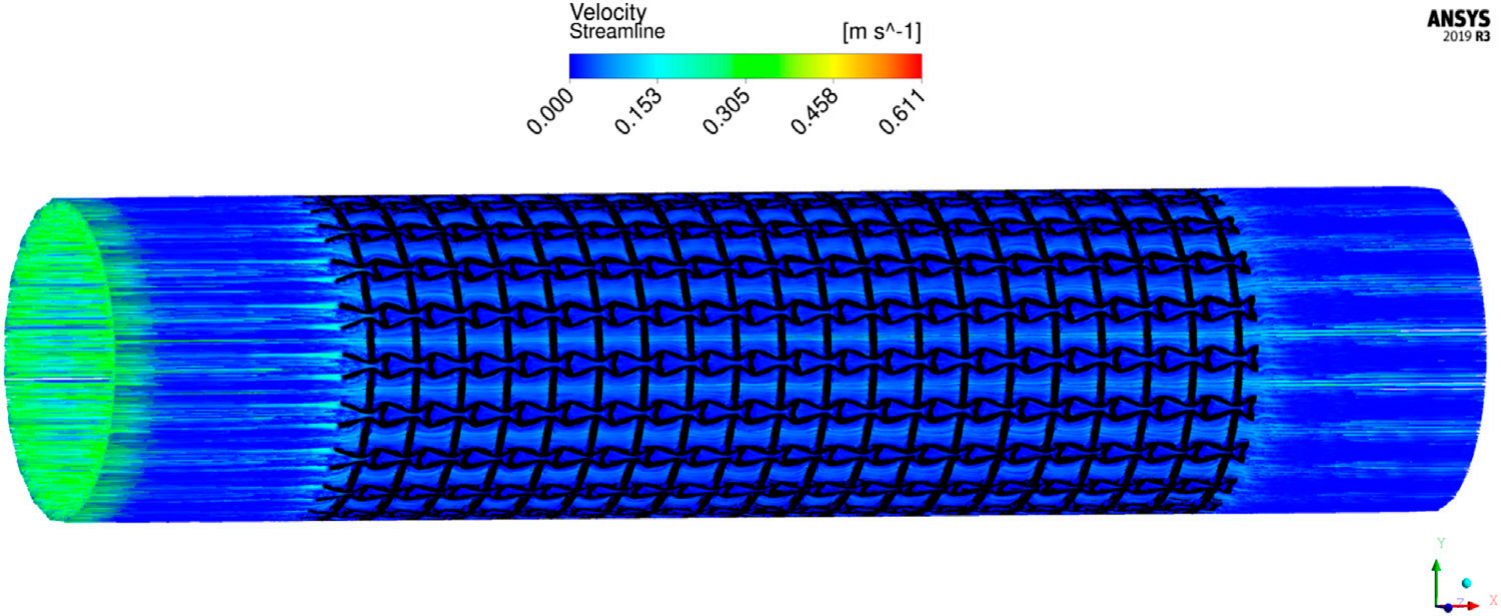
The streamlines of blood around the stent F-7.

The results of blood flow streamlines are shown in Figure 10, where the lower velocities are denoted with darker colours. In the figure, the maximum velocity of the flow is greater than the inflow condition, because the flow can be accelerated around the stent struts. Due to the applied no-slid boundary conditions, the minimum velocity is zero. The result shows that the velocities of the streamlines are affected by the stenting struts. Since relatively narrow spaces are formed between the horizontal struts in each unit cell, the blood flows are obstructed around these spaces and exhibits lower velocities. And the blood flows between each unit cell can maintain higher speeds due to fewer obstructions and larger fluid areas. Overall, blood flow can keep a high velocity, which can be demonstrated that many streamlines present light colours through the whole fluid region.

**FIGURE 11:**
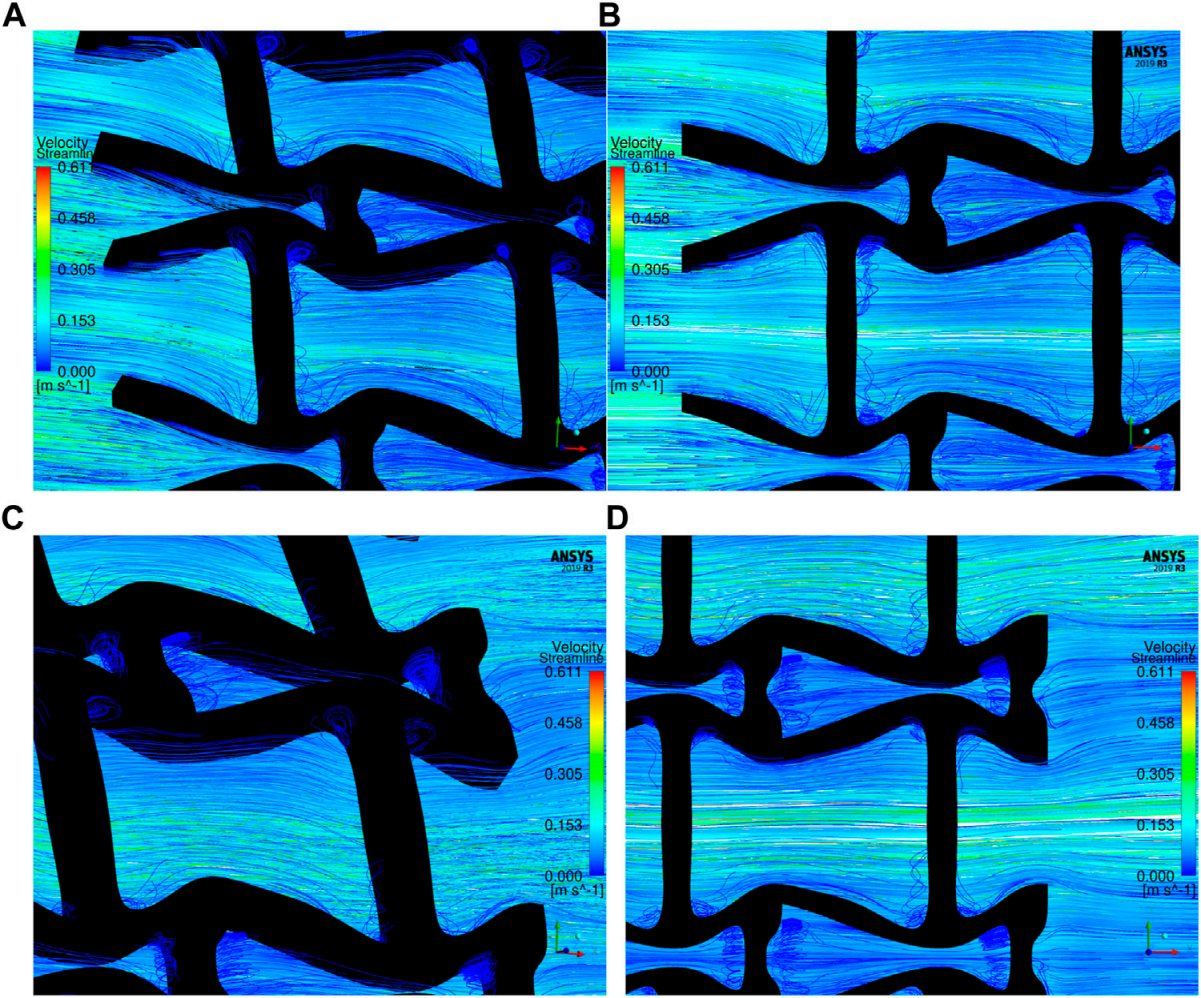
The streamlines of blood around the proximal and distal struts: **(A)** Proximal streamlines (Axonometric view); **(B)** Proximal streamlines (Side view); **(C)** Distal streamlines (Axonometric view); **(D)** Distal streamlines (Side view).

The overall influences of stenting obstructions can be evaluated by blood flow changes in the upstream side and downstream side of the stent. The streamlines in the proximal and distal struts are presented in Figure 11. In the figure, the fluid region around the stent can be divided into two different parts: one is the inner space of each individual cell, and the other is the connecting space between cells. When blood through these spaces, the flow can be accelerated around the struts and may lead to recirculation zones. In the connecting spaces of the proximal struts, the disturbances of the flow focus on the four corners and cause small recirculation zones. Also, a few vertical flows occur along the left struts in the connecting spacings. However, small recirculation zones are formed around the vertical struts in the inner spaces of proximal struts due to the narrow fluid spaces. Compared with the proximal struts, similar blood flow behaviours can be found around the distal struts but exhibit relatively smaller flow disturbances and recirculation zones. Although the recirculation zones may induce adverse effects, they only cover very few regions around the struts. Hence, the result shows that the optimization for the MFP can effectively reduce the vertical flow and the stenting obstructions to benefit blood flow.

**FIGURE 12:**
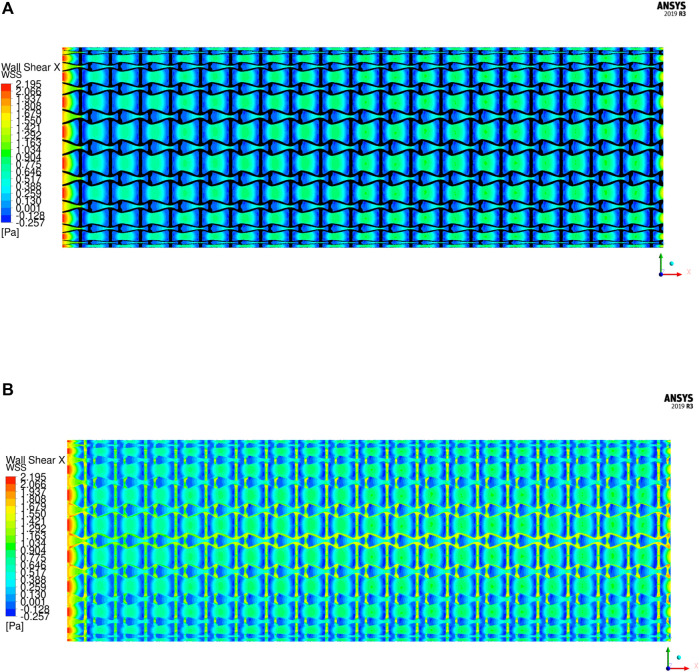
The WSS distributions of the stent: **(A)** Covered by the stent; **(B)** Without the stent.

The WSS is an important metric to quantify the hemodynamic impacts in stent implantation. The unusual WSS is often associated with adverse effects including ST (extremely high WSS) and ISR (extremely low WSS). When the magnitude of WSS is higher than 2.5 Pa increases the risk of ST, while lower than 0.5 Pa increases ISR incidence. Based on the flow direction, the WSS is mainly determined by the axial component, so the axial WSS is utilized to evaluate the stent. The axial WSS of the stent is illustrated in Figure 12. It can be found that the regions covered by the stent have higher WSS. In the results, the maximum axial WSS is 2.195 Pa, which is less than the high adverse stress 2.5 Pa and indicates a lower ST incidence. However, the regions surrounded by the stent struts exhibit lower and even negative axial WSS, which are recirculation zones. The same recirculation zones are also found in the streamline results. Among them, the connecting spaces between unit cells have higher WSS compared with their inner spaces due to larger fluid regions. The average WSS in most regions of the stent is around 0.9 Pa and greater than the lower bound of the threshold 0.5 Pa, but the areas close to the vertical struts exhibit smaller, even negative WSS. It is because the struts protrude into the artery lumen and separate the laminar flow, resulting in lower shear stress around the struts. This kind of effect is determined by the shape and size of the strut profiles and cannot be eliminated unless the stent is fully embedded into the artery walls. The regions with lower WSS cannot be completely avoided, but the impacted areas are small. Moreover, the WSS distribution of the stent is relatively uniform, which can avoid structural fatigue breakage to extend stent lifetime in use and is able to low occurrence of ST and ISR.

Hence, from the above numerical examples and discussions we only found limited disturbance in blood flow around the area of the stent, which indicates less obstructions of the newly designed stent to the blood flow. This shows the efficiency of the optimally designed structural architectures in stents.

## Conclusion

This paper has developed a family of auxetic architectures for stenting structures using a topological optimization approach and its associated numerical methods, which is especially beneficial to self-expanding stents. The new design combines the hemodynamic effect with auxetic structures to improve the stent performances particularly from both mechanical and hemodynamic aspects. Several numerical examples and their simulation results have shown the efficiency of the topologically optimized stenting architectures. The homogenized MFP can quantify the disturbances of the stenting microstructures to the blood flow, through which the reduction of the adverse blood flows can be transformed to the reduction of the obstructions of the stent to the blood flow. The obstructions have been found to associate with the material layouts of the microstructures. The fluent simulation results present fewer obstructions of the optimized stent to the blood flow and fewer adverse WSS distributions associated with ST and ISR risk factors. Hence, the newly developed auxetic architectures can benefit stenting performance by reducing occurrence of mechanical failures as well as the influence of adverse hemodynamics, which will help low the incidence of ST and ISR complications due to mechanical structural issues in stent implantation and treat heart disease in clinic practice.

In this study, it is noted that the pulsatile blood flow state is not considered in the design. Furthermore, this paper is focused on generic design of a new type of stenting architectures sharing common geometric profiles, not patient-specific and customised designs, through a topological optimisation method using idealized models. The success rate of PCI therapy has been significantly improved over the past, but the ST and ISR problems have not been completely resolved. Various factors may account for the incidence of ST and ISR in stent implantation. However, this work emphases the importance of mechanical structural failures and hemodynamics, rather than biomaterial, patient, clinical and operational aspects. Moreover, technical details for developing end-user products such as compressing stents into sheaths, injection and release of stents in implantation are outside of the scope of this paper.

## Data Availability

The original contributions presented in the study are included in the article/Supplementary Material, further inquiries can be directed to the corresponding author.
